# Structure and Properties of Porous Ti_3_AlC_2_-Doped Al_2_O_3_ Composites Obtained by Slip Casting Method for Membrane Application

**DOI:** 10.3390/ma16041537

**Published:** 2023-02-12

**Authors:** Egor Kashkarov, Maksim Krinitcyn, Adilzhan Dyussambayev, Alexey Pirozhkov, Maksim Koptsev

**Affiliations:** 1 School of Nuclear Science and Engineering, Tomsk Polytechnic University, 30 Lenina av., 634050 Tomsk, Russia; 2 Institute of Strength Physics and Materials Science SB RAS, 2/4, pr. Akademicheskii, 634055 Tomsk, Russia

**Keywords:** porous composites, alumina, mechanical properties, microstructure, ceramic supports

## Abstract

In the present work, porous composites were fabricated from pure Al_2_O_3_ and mixed Ti_3_AlC_2_/Al_2_O_3_ powder by slip casting and sintering. The effect of sintering temperature and different composition ratio on microstructure, phase composition, porosity and gas permeation flux of the fabricated materials was investigated. The microstructure and phase composition of the samples were analyzed by scanning electron microscopy and X-ray diffraction, respectively. The gas permeation experiments were performed using pure hydrogen at 0.1–0.9 MPa pressure. It is shown that a decrease in sintering temperature from 1500 to 1350 °C results in an increase in hydrogen permeation flux of the alumina from 5 to 25 mol/(m^2^ × s), which is due to higher pore size and overall porosity of the samples. Sintering of Ti_3_AlC_2_/Al_2_O_3_ powder mixtures leads to the formation of Al_2_O_3_, Al_2_TiO_5_ and TiO_2_ phases as a result of oxidation of the Ti_3_AlC_2_ phase, resulting in an increased pore size in the composites compared with pure alumina. The open porosity of composites increases from 3.4 to 40% with an increasing Ti_3_AlC_2_/Al_2_O_3_ ratio from 1/10 to 1/2, respectively. The composites with the highest porosity (40%) had a maximum permeation flux of 200 mol/(m^2^ × s). The changes in the bending strength of the alumina and composite samples, depending on the microstructure and porosity, were also discussed. The investigated composites are considered promising materials for hydrogen separation membrane supports.

## 1. Introduction

Currently, the issue of obtaining efficient and clean energy is very important. Hydrogen energy is one of the most promising areas for the development of alternative energy sources [1]. The commercial production of hydrogen usually results in low purity of hydrogen gas that cannot be used, for example, in fuel cells [2]. Therefore, different membrane systems are developed for hydrogen purification. The effective high-temperature membranes are made from palladium-based or other highly permeable metallic thin layers deposited on porous ceramic supports [3,4,5]. Ceramic supports should have high resistance to hydrogen embrittlement, suitable gas permeability and high mechanical strength [6].

Currently, one of the most effective supports is aluminum oxide due to low cost and chemical inertness to hydrogen and other gases [7,8]. The pores in aluminum oxide materials should be controlled to obtain good transportation properties for gas molecules diffused through the membranes [9]. MAX phases are a relatively new class of nanolaminated materials, generally described as M_n+1_AX_n_ (where M—transition metal, A—element of A group (mostly IIIA and IVA), X—carbon and/or nitrogen, n = 1–3). They are also promising as support material itself or in combination with other materials for supports and membranes due to their catalytic activity and combined properties of metals and ceramics, such as high thermal and electrical conductivities, high mechanical properties, high-temperature oxidation and thermal shock resistance [10,11,12,13,14]. Ti_3_AlC_2_ MAX phase and Al_2_O_3_ have the near-identical coefficients of thermal expansion, and Ti_3_AlC_2_/Al_2_O_3_ composites have superior spallation resistance under service conditions [15]. This makes it possible to protect composites from cracking and destruction during repeated heating/cooling cycles. Since one of the mechanisms of oxidation of the MAX phase is the formation of a thin layer of Al_2_O_3_, the use of the MAX phase in a composite with Al_2_O_3_ makes it possible to slow down the oxidation and form a strong bond between the particles of the MAX phase and Al_2_O_3_ as a result of oxidation [16,17,18,19]. In addition, the introduction of MAX-phase particles into the Al_2_O_3_ matrix increases the mechanical strength of the composites and the resistance to cracking [20,21]. The change in these and other properties directly depends on the microstructure and porosity of the material as well as its composition [22,23,24]. There are no experimental works on the microstructure, porosity and gas permeability of composites sintered from Al_2_O_3_ and Ti_3_AlC_2_ at different powder ratios. In this work, porous Al_2_O_3_ and composite samples with different Al_2_O_3_ and Ti_3_AlC_2_ powder ratios are obtained by slip casting and sintering. The influence of the Ti_3_AlC_2_/Al_2_O_3_ ratio on phase composition, microstructure, porosity and gas permeability of the obtained composites was investigated.

## 2. Experimental Details

### 2.1. Sample Preparation

The Ti_3_AlC_2_ MAX-phase powder (98% purity, d50 = 5 µm) was used in this work. As a source of Al_2_O_3_, waxed thermoplastic slip of corundum ceramics was used (VK-94.2 M7, the content of Al_2_O_3_ was not less than 94 wt.%). This material can be used to obtain materials with different porosity depending on the sintering temperature, and it has a relatively low sintering temperature as well as stability of the thermomechanical properties in a wide temperature range.

The plasticized slurry contains paraffin in an amount of 12 wt.%, which had to be removed to obtain a dry powder. The removal of the paraffin binder was ensured by sintering the samples at a temperature of 800 °C. The removal took place in several successive stages. The first stage is drying at a temperature of 90 °C for 10 h. The second stage is a slow heating up to 300 °C for 12 h (17.5 K/h), followed by heating up to 800 °C for 4 h (125 K/h) with isothermal holding at 800 °C for 2 h.

The obtained samples were cleaned from filling and ground in a Pulverisette 6 Planetary Mono Mill (Fritsch, Idar-Oberstein, Germany) in a zirconia drum with zirconia grinding bodies until the material completely passed through a sieve with a mesh size of 0.063 mm.

Ti_3_AlC_2_ powder was mixed with Al_2_O_3_ powder in mass ratios of 1/2, 1/4, 1/6, and 1/10, respectively. Mixing and homogenization of mixtures was carried out in a laboratory vibrating mill Pulverisette 23 (Fritsch, Germany) in a zirconia drum using zirconia grinding balls. The mass of the mixture for one mixing was 2 g, the oscillation frequency was 35 Hz, and the processing time was 5 min.

From the resulting mixtures, a composite was prepared with an alcohol solution of polyvinyl butyral binder (concentration—5%); the relative content of the binder in the press powder was 10 wt.%. Samples were made from composite on a laboratory press (LabTools, Saint-Petersburg, Russia) by uniaxial cold pressing (stainless steel die, diameter 12 mm); pressing pressure was 440 MPa. After that, the samples were dried to constant weight at a temperature of 70 °C.

The samples obtained were sintered at a temperature of 1350–1500 °C with a step of 50 °C in a shaft electric resistance furnace TK-27 1700Sh3F (Thermoceramics, Ekaterinburg, Russia) with lanthanum chromite heaters. The heating rate was 100 K/h, and the isothermal holding time was 2 h.

### 2.2. Characterization

The phase composition of the samples was analyzed by X-ray diffraction (XRD) using the XRD 7000S (Shimadzu, Tokyo, Japan) diffractometer equipped with a OneSight high-speed 1280-channel detector. The measurements were performed in Bragg–Brentano configuration using CuKα radiation (*λ* = 0.154 nm) at 40 kV and 30 mA. A spectrum was acquired for each of the samples at the following parameters: scanning step—0.0143 deg; sample scanning speed—10 deg/min; 2θ angle range—10–70 deg. The microstructure and elemental composition of the samples were analyzed by scanning electron microscopy (SEM) using the Vega 3 (TESCAN, Brno, Czech Republic) equipped with an energy-dispersive X-ray spectroscopy (EDS) attachment.

The density of samples was determined using a hydrostatic weighing method (Archimedes method) in kerosene. The mechanical strength was measured by a three-point bending test performed using the Gotech Al-7000M machine (GoTech, Taichung City, Taiwan). For bending testing, 2 mm × 2 mm × 18 mm samples were cut from the specimens using a diamond disk. The Gotech data acquisition software was used to trigger the minimum load (0.1 N) on the sample and to start a data collection. The acquisition frequency was 124 Hz. The loading rate was set up as 0.5 mm/min according to the ASTM standard (E290). The span distance was equal to 16 mm.

### 2.3. Hydrogen Permeability Measurement

**Figure 1:**
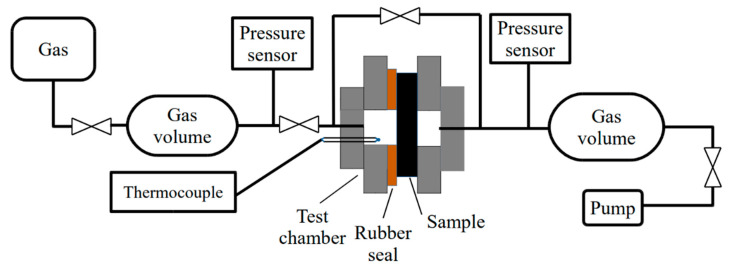
Schematic representation of the gas permeability cell.

Hydrogen permeability tests were carried out using the Gas Reaction Automated Machine (GRAM, TPU, Tomsk, Russia) equipped with a gas permeation cell. For the hydrogen permeability test, the samples were placed in a test cell with a rubber sealing gasket. The active surface area of the specimens was 1.9 × 10^−5^ m^2^. The cell was evacuated to a residual pressure of 1 × 10^−3^ Pa on both sides of the sample. The tests were performed at room temperature (25 °C). On the gas supply side, the hydrogen pressure was set to 0.1–0.9 MPa. The gas pressure drop was measured on the precalibrated gas supply volume. The schematic diagram of the setup is shown in Figure 1.

The main characteristics of the supports and membranes are gas flow and permeability. Flux (Φ) is the total transport of a substance across a membrane and can be expressed as mass or molar concentration per unit time per unit area. Permeability (J) is defined as flow per unit pressure difference between the inlet and outlet side of the membrane. This is shown in the following equation:

$$j=\Phi \times \frac{d}{\Delta P},$$

where Φ—gas flow (mol/(m^2^ × s)), J—gas permeability (mol/(m × s × Pa)), d—sample thickness (m), ΔP—pressure difference at the inlet and outlet of the sample (Pa).

## 3. Results and Discussion

### 3.1. Microstructure and Phase Composition

**Figure 2:**
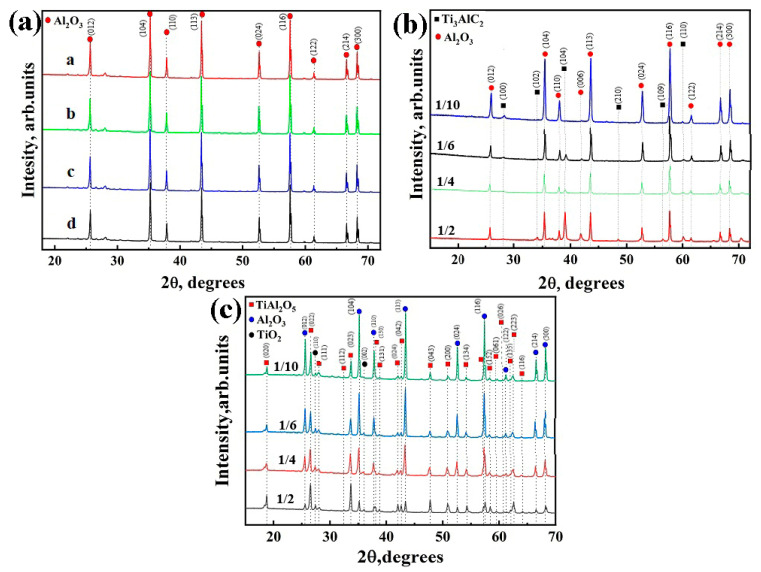
XRD patterns of (**a**) porous Al_2_O_3_ at different sintering temperatures: a—1500 °C, b—1450 °C, c—1400 °C, d—1350 °C; (**b**) green bodies before sintering and (**c**) porous composites obtained from Ti_3_AlC_2_/Al_2_O_3_ powders.

The XRD patterns for the green bodies and sintered samples are shown in Figure 2. For sintered aluminum oxide samples, only the α-Al_2_O_3_ phase was found at all the sintering temperatures (Figure 2a). The as-received green bodies of composite samples are represented by the α-Al_2_O_3_ and hexagonal closed-packed Ti_3_AlC_2_ phases (Figure 2b). The increase in intensities of the corresponding peaks of the Ti_3_AlC_2_ phase indicates its higher volume content at a higher Ti_3_AlC_2_/Al_2_O_3_ powder ratio. After sintering, the Ti_3_AlC_2_ phase undergoes oxidation during sintering of Ti_3_AlC_2_/Al_2_O_3_ composites (Figure 2c), which resulted in the formation of Al_2_TiO_5_ and TiO_2_ oxide phases [13,14,15,16]. The results of phase composition analysis are presented in Table 1. Since no secondary phases were observed for pure aluminium and the lattice parameters did not change after the sintering, the data for these samples are not included in Table 1. It can be seen that the content of the oxide Al_2_TiO_5_ and TiO_2_ phases in the sintered composite samples increases with increasing Ti_3_AlC_2_/Al_2_O_3_ powder ratio.

The microstructural analysis of the sintered Al_2_O_3_ porous ceramics showed that pore size and overall porosity decrease with increasing sintering temperature. The measured open porosity by the Archimedes method decreased from 37 to 21% when the sintering temperature increased from 1350 to 1500 °C, respectively (Table 2). At the same time, the apparent density of Al_2_O_3_ samples increased from 2.31 to 3.07 g/cm^3^, while the average pore size decreased from 1.25 to 0.5 µm.

**Figure 3:**
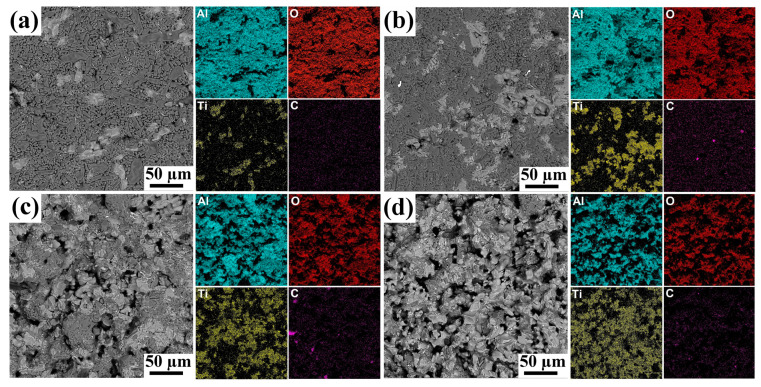
SEM images and corresponding EDS elemental maps for porous composites obtained from Ti_3_AlC_2_/Al_2_O_3_ mixture: 1/10 (**a**), 1/6 (**b**), 1/4 (**c**) and 1/2 (**d**).

**Figure 4:**
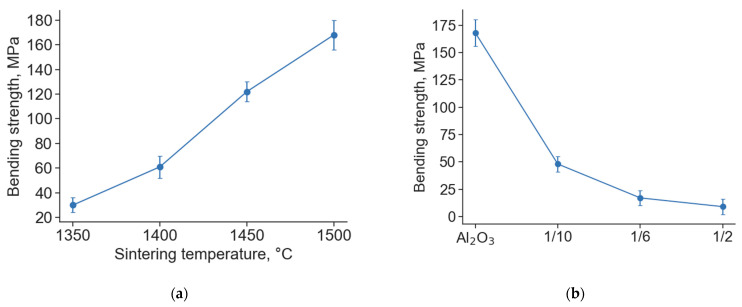
Bending strength of Al_2_O_3_ samples after sintering in air at different temperatures (**a**) and bending strength of samples of pure Al_2_O_3_ and mixtures of Ti_3_AlC_2_/Al_2_O_3_ after sintering in air at 1500 °C (**b**).

The addition of the MAX phase leads to the formation of more open pores in the composites (Figure 3). The average pore size increased from ~0.5 to 6.5 µm with increasing powders ratio from 1/10 to 1/2. It should be noted that the pores have rounded and elongated shapes. The presence of the MAX phase changes the sintering kinetics as well as the microstructure of the composite samples (Figure 4). In addition to the sintering process, the decomposition and oxidation of the MAX phase proceed at high temperatures. The formation of the Al_2_TiO_5_ phase from titanium and aluminum in oxygen (ΔG = −2640.1 + 0.5T kJ/mol) is more likely than the formation of Al_2_O_3_ (ΔG = −1692.5 + 0.33T kJ/mol) and TiO_2_ (ΔG = −940.4 + 0.181T kJ/mol) [25]. Thus, during the sintering processes, energy is spent on the decomposition of the MAX phase, as well as on other processes, which deintensifies the sintering kinetics. The formation of new phases during sintering of a mixture of powders with different thermal expansion coefficients will also prevent the formation of strong bonds between the particles. In addition, the formed particles are different in size, shape and chemical nature, which also affects the sintering kinetics and the final microstructure and porosity of the composites. 

The microstructure of the composites is represented by the alumina phase (dark contrast) and TiO_2_ + Al_2_TiO_5_ phase (bright contrast) (Figure 3). EDS analysis revealed that the increase in Ti_3_AlC_2_ content results in a more homogeneous distribution of the oxide phases and a more uniform distribution of pores in the composites. The content of the oxide phases formed from MAX-phase oxidation increases with the Ti_3_AlC_2_/Al_2_O_3_ ratio that is well correlated with XRD data. The distribution of these oxides is more uniform at higher ratios of Ti_3_AlC_2_ to alumina powders. The open porosity increases from 3.4 to 40% with an increase in the powder ratio from 1/10 to 1/2, respectively (Table 2). At the given sintering temperature, a more uniform porous structure was formed only at 1/4 and 1/2 powder ratios. 

The results showed that variation of the Ti_3_AlC_2_/Al_2_O_3_ ratio makes it possible to control the porosity of the resulting samples, ranging from relatively dense to highly porous composites.

### 3.2. Mechanical Properties

Figure 4 shows the results of bending strength measurements for both pure Al_2_O_3_ (sintered at different temperatures) and composite samples obtained from Ti_3_AlC_2_/Al_2_O_3_ mixtures.

For pure Al_2_O_3_ samples, the bending strength increases from 25 to 165 MPa with an increasing sintering temperature from 1350 to 1500 °C, respectively (Figure 4). We used a commercial Al_2_O_3_ powder for which the manufacturer recommended a sintering temperature of 1580 ± 30 °C to obtain dense material. Lower sintering temperatures were used to obtain a microporous structure of the material and to analyze the influence of its porosity on the bending strength. It was demonstrated that the strength of all investigated Al_2_O_3_ samples remains at a sufficiently high level compared with alumina support materials with similar density produced in other works [26,27,28]. The bending strengths of the alumina samples with acceptable porosity for support material (obtained at 1350 and 1400 °C) were 25 and 60 MPa, respectively.

The bending strength of the composite samples decreased from 50 to 10 MPa with increasing Ti_3_AlC_2_/Al_2_O_3_ powder ratios from 1/10 to 1/2, respectively (Figure 4b). The analysis of microstructure and porosity showed that the porosity of the composites increased with the Ti_3_AlC_2_/Al_2_O_3_ powder ratio. Thus, it is assumed that the main reason for the reduction in strength is the change in porosity of the composite samples. In addition, at comparable porosity values, the strength of the composites is lower than that of pure alumina samples. This indirectly confirms the weaker bonding between the particles and the possible internal stresses in the composite samples.

### 3.3. Hydrogen Permeability

**Figure 5:**
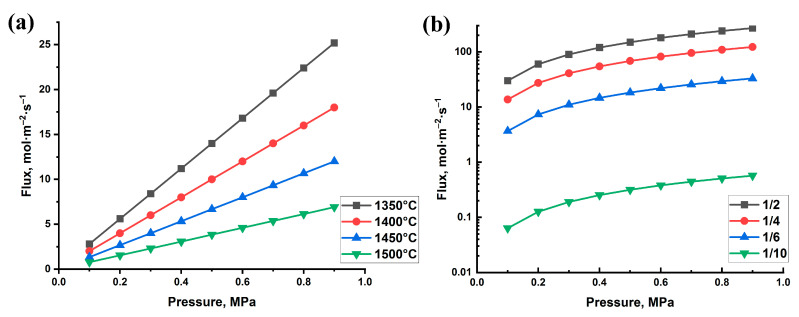
Hydrogen permeation flux depending on pressure for the porous samples obtained from pure Al_2_O_3_ (**a**) and Ti_3_AlC_2_/Al_2_O_3_ powder mixtures (**b**).

The permeability tests show the dependence between hydrogen permeation flux through the samples and gas pressure (Figure 5). The results showed near linear dependence of hydrogen flux on pressure for all the alumina samples (Figure 5a). The flow rate increases from 5 to 25 mol/(m^2^ × s) (0.9 MPa pressure) when the sintering temperature increases from 1350 to 1500 °C, respectively. Thus, the increase in porosity of Al_2_O_3_ results in a fivefold higher gas flow through the samples (Figure 5a). It can be seen that the increase in the Ti_3_AlC_2_/Al_2_O_3_ ratio and the corresponding porosity result in higher hydrogen flux through the samples. Similar dependence between the flux and gas pressure was found for all the samples in the investigated hydrogen pressure range. The hydrogen flux for the sample with 1/10 ratio is in the range of 0.06–0.4 mol/(m^2^ × s), which is relatively low for support materials. The maximum flow of 30–200 mol/(m^2^ × s) was achieved for the sample with the highest porosity (1/2 ratio), which is suitable for ceramic-based supports developed for gas separation membranes [29].

The flux of hydrogen through a thin film of palladium (0.117–50 µm) at a temperature of 500 °C with a pressure difference of 0.2 MPa and a permeability of 1.9 × 10^−8^ (mol/(m × s × Pa)) will be 0.17–72 mol/(m^2^ × s) [30,31]. When the hydrogen flux through the ceramic substrate is 1–80 mol/(m^2^ × s) at a thickness of 3 mm, the hydrogen flux will be limited only by a dense layer of palladium. In [32], the hydrogen flux through the 1 mm thick porous Al_2_O_3_ substrate embedded in a porous stainless steel base was 0.002 mol/(m^2^ × s). Checchetto et al. [33] used Al_2_O_3_ with a thickness of 60 μm; the hydrogen flux was 0.15 mol/(m^2^ × s) at a pressure difference of 0.1 MPa. Thus, the fabricated alumina and composite samples have high hydrogen permeability at a thickness of 2–3 mm and good mechanical strength.

## 4. Conclusions

Porous composites were fabricated from pure alumina and mixed Ti_3_AlC_2_ and Al_2_O_3_ powders by slip casting. The influence of alumina sintering temperature and Ti_3_AlC_2_/Al_2_O_3_ powder ratio on microstructure, porosity and gas permeability of the fabricated porous materials was analyzed. The following conclusions were made:

1. The decrease in sintering temperature from 1500 to 1350 °C results in an increase in the porosity of alumina samples from 21 to 37% and an increase in their pore size. The hydrogen permeation flux increases up to 25 mol/(m^2^ × s) for alumina samples sintered at 1350 °C.
2. The addition of Ti_3_AlC_2_ to alumina powder leads to the formation of composite oxide ceramics with higher pore size. The phase composition of the sintered composites is represented by Al_2_O_3_, TiO_2_ and Al_2_TiO_5_ phases and is caused by oxidation of the Ti_3_AlC_2_ phase. The distribution of the oxides formed from the Ti_3_AlC_2_ phase is more uniform at higher ratios of MAX phase to alumina powders.
3. The porosity of composites increases from 3.4 to 40% with increasing the Ti_3_AlC_2_/Al_2_O_3_ powder ratio from 1/10 to 1/2, respectively. The increase in porosity of composites provides better gas permeability. The maximum hydrogen flux up to 200 mol/(m^2^ × s) was achieved for the sample with the highest porosity of 40%.
4. The bending strength of the sintered samples decreases with the addition of MAX phase to alumina powder but increases the gas permeability. All the fabricated materials demonstrate relatively high bending strength, which was 25 and 10 MPa for highly porous alumina (37%) and composite (40%) materials. The fabricated materials can be used for membrane support application.

## Figures and Tables

**Table 1:** Phase composition of the composite samples calculated from XRD data.

Sample Name	Phase	Content, %	Lattice Parameters, Å	Phase	Content, %	Lattice Parameters, Å
	Before Sintering			After Sintering		
Composite 1/2	Al_2_O_3_	62	a = 4.763 c = 13.005	Al_2_O_3_	16	a = 4.752
						c = 12.979
	Ti_3_AlC_2_	38	a = 3.087 c = 18.588	TiO_2_	5	a = 4.588
						c = 2.966
				TiAl_2_O_5_	79	a = 3.586
						b = 9.430
						c = 9.641
Composite 1/4	Al_2_O_3_	81	a = 4.761 c = 12.998	Al_2_O_3_	34	a = 4.761
						c = 12.999
	Ti_3_AlC_2_	19	a = 3.075 c = 18.560	TiO_2_	6	a = 4.598
						c = 2.966
				TiAl_2_O_5_	60	a = 3.590
						b = 9.449
						c = 9.659
Composite 1/6	Al_2_O_3_	88	a = 4.762 c = 13.001	Al_2_O_3_	45	a = 4.760
						c = 12.997
	Ti_3_AlC_2_	12	a = 3.080 c = 18.569	TiO_2_	7	a = 4.597
						c = 2.962
				TiAl_2_O_5_	48	a = 3.590
						b = 9.449
						c = 9.662
Composite 1/10	Al_2_O_3_	93	a = 4.762 c = 12.999	Al_2_O_3_	49	a = 4.761
						c = 12.996
	Ti_3_AlC_2_	7	a = 3.077 c = 18.565	TiO_2_	4	a = 4.582
						c = 2.959
				TiAl_2_O_5_	47	a = 3.591
						b = 9.451
						c = 9.659

**Table 2:** The apparent density, open porosity and water absorption of the samples measured by the Archimedes method.

Sample	Water Absorption, %	Porosity, %	Open Porosity, %	Apparent Density, g/cm^3^	Average Pore Size, µm
Composite 1/2	15.7	-	40.1	2.01	6.5
Composite 1/4	7.8	-	27.2	2.76	4
Composite 1/6	3.4	-	14.0	3.27	3.5
Composite 1/10	0.8	-	3.4	3.54	<0.5
Al_2_O_3_ 1350 °C	12.7	41.3	37.1	2.32	1.25
Al_2_O_3_ 1400 °C	10.8	37.7	33.5	2.46	0.85
Al_2_O_3_ 1450 °C	8.2	31.1	28.3	2.72	0.65
Al_2_O_3_ 1500 °C	5.5	22.3	21.4	3.07	0.5

## Data Availability

Data are contained within the article.
